# Real-time monitoring of increases in restlessness to assess idiographic risk of recurrence of depressive symptoms

**DOI:** 10.1017/S0033291722002069

**Published:** 2023-08

**Authors:** Arnout C. Smit, Evelien Snippe

**Affiliations:** 1 Department of Psychiatry, Interdisciplinary Center Psychopathology and Emotion regulation, University of Groningen, University Medical Center Groningen, Groningen, The Netherlands; 2 Faculty of Behavioral and Movement Sciences, Clinical Psychology, VU Amsterdam, Amsterdam, The Netherlands

**Keywords:** Anxiety, early detection, early warning signals, experience sampling method, mobile monitoring, prediction tool, replicated single-subject design

## Abstract

**Background:**

This confirmatory study aimed to examine whether we can foresee recurrence of depressive symptoms using personalized modeling of rises in restlessness.

**Methods:**

Participants were formerly depressed patients (*N* = 41) in remission who (gradually) discontinued antidepressants. Participants completed five smartphone-based Ecological Momentary Assessments (EMA) a day, for a period of 4 months, yielding a total of 21 180 observations. Statistical Process Control by means of Exponentially Weighted Moving Average (EWMA) control charts was used to detect rises in the EMA item ‘I feel restless’, for each individual separately.

**Results:**

An increase in restlessness was detected in 68.3% of the participants with recurring depressive symptoms, and in 26.3% of those who stayed in remission (Fisher's exact test *p* = 0.01, sensitivity was 68.3%, specificity was 73.7%). In the participants with a recurrence and an increase in restlessness, this increase could be detected in the prodromal phase of depression in 93.3% of the cases and at least a month before the onset of the core symptoms of depression in 66.7% of the cases.

**Conclusions:**

Restlessness is a common prodromal symptom of depression. The sensitivity and specificity of the EWMA charts was at least as good as prognostic models based on cross-sectional patient characteristics. An advantage of the current idiographic method is that the EWMA charts provide real-time personalized insight in a within-person increase in early signs of depression, which is key to alert the right patient at the right time.

## Introduction

Depression is often preceded by a prodromal phase; a period with signs and symptoms that anticipate the onset of a fully manifested disorder (Fava & Kellner, 1991; Fava & Tossani, 2007). Symptoms of anxiety have been reported retrospectively as common prodromal symptoms (Hetrick et al., 2008; Pede, Jaiswal, & Sawant, 2017), and have been shown to predict future depressive symptoms (Fava & Tossani, 2007; Hettema, Kuhn, Prescott, & Kendler, 2006; Jacobson & Newman, 2017). A systematic review found that symptoms of anxiety, such as feeling anxious, tension, and irritability, were among the most commonly reported prodromal symptoms of depression (Benasi, Fava, & Guidi, 2021). If symptoms of anxiety precede depression, it would be useful to monitor these symptoms in order to treat them and thereby prevent manifestations of the core symptoms of depression (Jacobson & Newman, 2017). However, it has yet to be examined whether real-time monitoring of symptoms of anxiety in individual patients would indeed reveal an increase in anxiety symptoms preceding the onset of depressive symptoms.

To monitor symptoms of anxiety and detect symptom change in time, frequent symptom observations and within-person modeling of symptom change is needed. Although both clinicians and researchers have advocated for the use of personalized models (Barlow & Nock, 2009; Bos, Snippe, Bruggeman, Wichers, & van der Krieke, 2019; Fisher & Boswell, 2016; Molenaar, 2004; Piccirillo & Rodebaugh, 2019; Wright & Woods, 2020; Zuidersma et al., 2020), idiographic methods that model mean changes in symptoms over time are not commonly used in the field of psychiatry and clinical psychology (Wright & Woods, 2020). Moreover, methods that can detect mean symptom change in real-time have almost never been applied. Whereas many studies have aimed to predict recurrence of depression based on static differences between individuals (e.g. Berwian, Walter, Seifritz, & Huys, 2017; Brouwer, Williams, Forand, DeRubeis, & Bockting, 2019; Buckman et al., 2018; Deng et al., 2018; Hardeveld, Spijker, De Graaf, Nolen, & Beekman, 2013; Ten Have et al., 2018; van Loo, Aggen, Gardner, & Kendler, 2018; Wojnarowski, Firth, Finegan, & Delgadillo, 2019), only a few studies have examined within-person symptom change over time as an indicator of future depressive symptoms (e.g., Cho et al., 2020; Kunkels et al., 2021; Wichers, Groot, & Psychosystems, 2016; Wichers, Smit, & Snippe, 2020).

A pilot study of Smit, Snippe, and Wichers (2019) provided first evidence of the potential usefulness of applying an idiographic method to detect within-person increases in the symptom ‘restlessness’ to foresee depression. This pilot study focused on restlessness as it is a defining symptom of anxiety and because the two participants of this study who experienced recurrence of depressive symptoms both mentioned that they had noticed an increase in restlessness before the onset of the core symptoms of depression. Restlessness may be a sign of sympathetic over-activation and hyperarousal, which may play a role in depression (Blake, Trinder, & Allen, 2018; Schiweck, Piette, Berckmans, Claes, & Vrieze, 2019) and the development of associated symptoms such as sleep problems and rumination (Blake et al., 2018).

The pilot study of Smit et al. (2019) examined whether restlessness increased before recurrence of depression by assessing restlessness three times a day using Ecological Momentary Assessment (EMA) for 95–183 days in seven formerly depressed patients who tapered their antidepressant medication. Using Exponentially Weighted Moving Average (EWMA) control charts, a type of Statistical Process Control charts (see e.g. Montgomery, 2012), an increase in mean levels of restlessness was detected more than 2 months before the onset of depressive symptoms in both patients with a recurrence of depressive symptoms. No significant rises in restlessness were found in all five participants that did not experience a recurrence of depressive symptoms.

As these results were promising, the current confirmatory study applies the same method in a much larger sample of formerly depressed patients in remission who discontinue their antidepressants. We hypothesize that rises in restlessness indicate a within-person increase in the risk for the recurrence of depression. Using a replicated single-subject design, the current study aims to examine whether rises in mean levels of restlessness can be detected in real-time more frequently in patients with recurrence of depressive symptoms than in patients that remain in remission. It is also examined if rises in mean levels of restlessness occur before symptom recurrence, and how long before recurrence increases in restlessness can be detected.

## Methods

### Participants

Participants were formerly depressed individuals in remission who discontinued their antidepressant medication and who participated in the TRANS-ID Tapering study (Smit, Helmich, Snippe, & Wichers, 2020; Smit, Snippe, Bringmann, Hoenders, & Wichers, submitted). Inclusion criteria for participating in the TRANS-ID Tapering study were: having made a shared decision with a mental health care provider to (gradually) discontinue antidepressant medication until a planned dosage of 0 mg, having made a tapering scheme or plan with their mental health care provider, age ⩾18, and fulfil the criteria of a past depressive episode according to DSM-IV criteria. Exclusion criteria were current depressive episode, bipolar disorder or psychotic disorder according to the DSM-IV criteria, reported diagnosis of a personality disorder, start of any other antidepressant treatment, and inability to work with a smartphone.

**Fig. 1.**
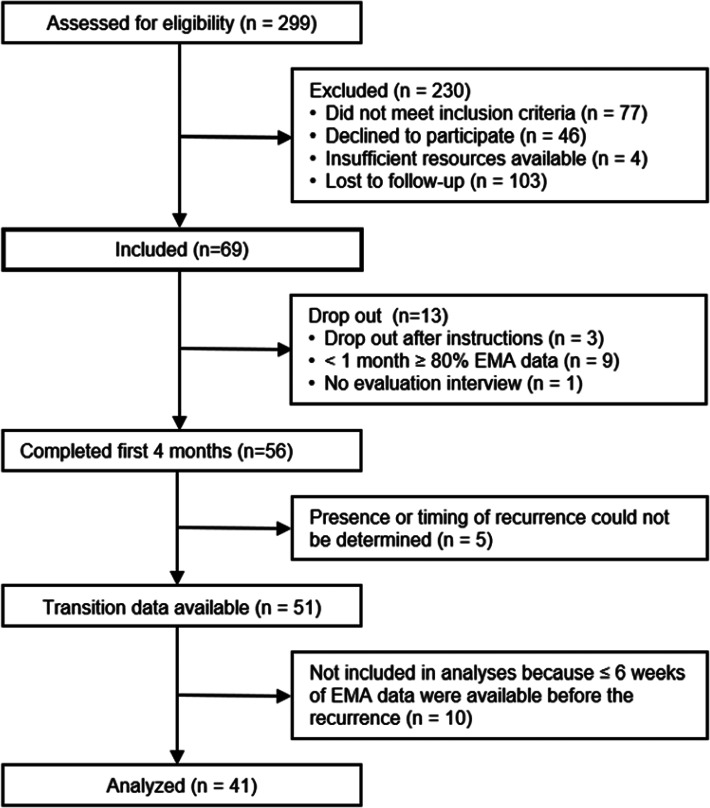
Flowchart of inclusion of participants.

Of the 299 participants who were assessed for eligibility, 69 patients meeting these criteria were included in the original study (see flowchart in Fig. 1). An additional 28 participants were excluded from the analyses in the current study because of the following reasons: <1 month of at least 80% EMA data, no completion of the evaluation interview, presence, absence, or timing of recurrence of depressive symptoms could not be determined, <6 weeks of EMA data before the recurrence (which is needed to obtain sufficient data for detecting increases in restlessness *before* the recurrence). The final sample consisted of 22 participants with depressive symptom recurrence and 19 participants that stayed in remission (*N* = 41).

### Procedures

Participants were recruited through advertisements distributed through social media and through a local pharmacy (Regenboog Apotheek) that offers antidepressant tapering strips (Groot & van Os, 2018). All participants provided written informed consent. The current study was approved by the Medical Ethical Committee of the University Medical Center Groningen. A study protocol of the TRANS-ID Tapering study (Smit et al., 2020) and an *a priori* analysis plan of the current study (Smit & Snippe, 2021) were published on the Open Science Framework (OSF).

**Fig. 2.**
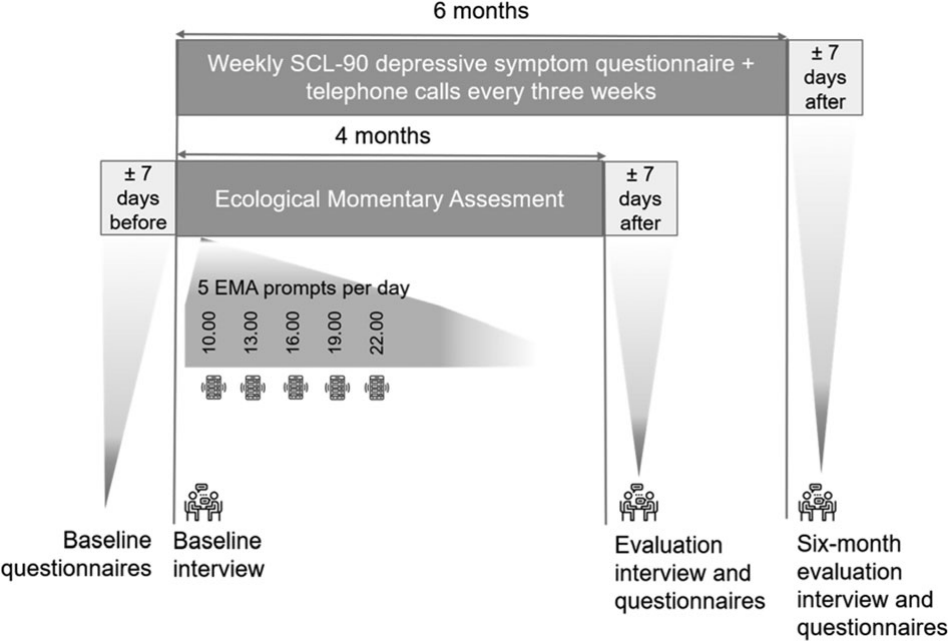
Design of the TRANS-ID Tapering study (EMA part). *Note:* EMA, Ecological Momentary Assessment. The heart rate variability and actigraphy assessments are not shown in the figure as these were not part of the current study.

The data collection started 1 month before planned full antidepressant discontinuation to a dosage of 0 mg (the discontinuation scheme differed between individuals). EMA was used to collect data on momentary experiences five times a day for 4 months, thus covering 1 month of tapering and 3 months after planned discontinuation (see for an overview of the design Fig. 2). In addition, participants completed weekly questionnaires including the depression subscale of the Symptom Checklist-90 (SCL-90) (Derogatis & Cleary, 1977) during these 4 months, and during the 2 months directly after. Patients participated in a semi-structured evaluation interview after 4 months and after 6 months. When participants completed at least 80% of the questionnaires, they were financially compensated. The 41 included participants completed a median of 540 EMA questionnaires (range 220–609), corresponding with a median compliance of 87.6%.

### Materials

#### Restlessness

Restlessness was measured with the EMA item ‘I feel restless’ on a visual analog scale ranging from ‘not at all’ to ‘very much’. This question was part of an EMA questionnaire that was filled out five times a day on a smartphone for 4 months.

#### Transitions toward higher levels of depressive symptoms

Presence or absence of recurrence of depressive symptoms was based on (1) a statistically reliable increase (⩾8.5 points) in depressive symptoms measured by the SCL-90 depressive symptom subscale (Derogatis & Cleary, 1977) compared to the first 2 weeks of the study, based on the Reliable Change Index (Jacobson & Truax, 1991), (2) whether this reliable increase persisted for at least three consecutive weeks, and/or treatment was started or increased, and/or tapering was interrupted, and (3) a consensus rating of a clinically meaningful increase in depressive symptoms made by three independent raters based on participants' self-reports of the first noticed meaningful increases in depressive symptoms. Information from (a) open text fields in daily and weekly questionnaires, (b) emails and telephone calls, and (c) the semi-structured evaluation interviews was used for the consensus rating. For the specific computation of the Reliable Change Index and the rating guide for consensus rating of clinically meaningful change, see Smit et al. (submitted) and Smit et al. (2020).

### Statistical analyses

First, the mean of restlessness on each day was computed for each participant, in order to handle autocorrelation (Carey, Parker, Robertson, Misbach, & Fisher, 2003; Schat, Tuerlinckx, Smit, De Ketelaere, & Ceulemans, 2021). A recent simulation study demonstrated using day averages reduced the autocorrelation in EMA data, and showed that there was no benefit of reducing the autocorrelation further (Schat et al., 2021). For each participant, the first 28 days (yielding a total of 140 beeps) were used as baseline period (phase I) in order to construct the personalized control limits for the EWMA, as this period includes sufficient observations and covers the period before the medication dosage was set to 0. Although there is little empirical evidence to guide the optimal number of observations in phase I, Smit et al. (2019) obtained good results with a phase I period of 100 observations. The other 3 months (phase II) were used to examine whether the EWMA of the day average of restlessness would exceed the personalized Upper Control Limit (UCL), signaling a significant increase in restlessness compared to phase I. The EWMA is a weighted mean of current and past observations and was computed as follows:
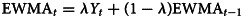


*Y_t_* represents restlessness at time *t*, EWMA_0_ is set to the mean of phase I, and lambda (*λ*) determines how much weight is given to the current observation. The EWMA control charts were run using *λ* = 0.1, since this has been shown to perform well when using day averages of EMA data (Schat et al., 2021). The UCL was computed as follows:
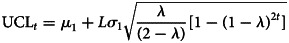
where *μ*_1_ is the mean of the phase I, *σ*_1_ is the standard deviation of phase I, *t* is the day number, and where *L* determines the width of the control limits. *L* was set to 2.56, corresponding with an average in-control run length (ARL_0_) of 318 days^†^^1^. The ARL_0_ represents the average number of days before a false positive occurs, which is ~10 months (i.e. 318 days) in the current study. Outliers (data points >3*σ*_1_ from *μ*_1_) were Winsorized to reduce the probability of false positives caused by outliers while having a smaller effect on the probability of detecting true structural changes. For more information about the EWMA procedure, including the derivation of the formulas above, see Montgomery (2012).

The percentage of true positives (sensitivity) and percentage of false positives (1-specificity) were computed. In a first analysis, we defined the number of true positives as the number of participants with recurrence of depression for which restlessness increased at any point in time. In a second analysis, we defined the number of true positives as the number of participants with recurrence of depression for which restlessness increased *before* the first day recurrence was signaled. In addition, the positive predictive value [PPV = true positives/(true positives + false positives)] and the negative predictive value [NPV = true negatives/(true negatives + false negatives)] were computed. Finally, the time between an increase in restlessness and depressive symptom recurrence was computed for each individual.

### Sensitivity analyses

The results are also reported for a restricted sample in which the participants with a transition after the 4-month ESM period were excluded (*N* = 8). As a second sensitivity analysis, the EWMA charts were re-run without Winsorizing the data. Furthermore, the EWMA charts were re-run using the raw data of the item ‘I feel restless’ (instead of using the day averages). This was done to make sure that differences in the results between the current study and the previous study of Smit et al. (2019) are not the result of this method artefact. To mimic the tuning choices of Smit et al. (2019), *L* was set to 3 and *λ* was set to 0.05. We controlled for autocorrelation at lag 1 if a model including autocorrelation at lag 1 had a lower AIC than a model in which autocorrelation was not modeled in the phase I data of that participant. Outliers (data points >3*σ*_1_ from *μ*_1_) were Winsorized.

## Results

### Sample characteristics

Table 1 provides an overview of sample characteristics of the participants. The total sample mostly represented participants who were female, highly educated, in a romantic relationship, and used tapering strips to taper antidepressant medication. Post-hoc analyses showed that there were no group differences in baseline characteristics that have been shown to predict recurrence of depression (Buckman et al., 2018; Klein, Holtman, Bockting, Heymans, & Burger, 2018) between patients who experienced a recurrence of depressive symptoms and patients who remained in remission; Welch's *t* tests revealed no difference between the two groups regarding the number of previous discontinuation attempts (*t* = 1.30, *p* = 0.20) and the SCL-90 depression score at baseline (*t* = 0.36, *p* = 0.72). The average restlessness score during phase I (*t* = 0.18, *p* = 0.86) also did not differ between the patients with a recurrence and patients who stayed in remission.

**Table 1.** Sample characteristics at baseline

Baseline characteristics	Recurrence (*N* = 22), [% (*N*)]/[M (s.d.)]	Remission (*N* = 19), [% (*N*)]/[M (s.d.)]
Age	48.4 (14.2)	44.3 (11.5)
Female	86.4% (19)	78.9% (15)
Education		
Primary/lower vocational school	0.0% (0)	0.00% (0)
Secondary/advanced vocational school	0.0% (0)	26.3% (5)
Higher education/university	100% (22)	73.7% (14)
Hours of paid work per week	16.3 (15.0)	20.8 (15.0)
In a romantic relationship	81.8% (18)	78.9% (15)
Type of antidepressant medication		
Venlafaxine	40.9% (9)	36.8% (7)
Paroxetine	22.7% (5)	5.3% (1)
Sertraline	13.6% (3)	5.3% (1)
Fluoxetine	4.5% (1)	15.8% (3)
Citalopram	9.1% (2)	26.3% (5)
Escitalopram	4.5% (1)	5.3% (1)
Clomipramine	4.5% (1)	0.0% (0)
Bupropion	0.0% (0)	5.3% (1)
Used tapering strips	86.4% (19)	73.7% (14)
Receives psychological treatment	41.2% (7)†	29.4% (5)†
Tried tapering before	76.5% (13)†	52.9% (9)†
*N* discontinuation attempts	1.5 (1.4)†	0.9 (1.2)†
Baseline SCL-90 depression	29.0 (9.9)	28.0 (8.5)
Restlessness during phase I	26.2 (10.2)	25.6 (12.2)

*Note:* † A small subset of participants did not fill out the questionnaire regarding psychological treatment and previous discontinuation attempts, meaning that cells indicated with a † are based on 17 participants that experienced recurrence, and 17 participants that remained in remission.

### Changes in restlessness at the individual level

**Fig. 3.**
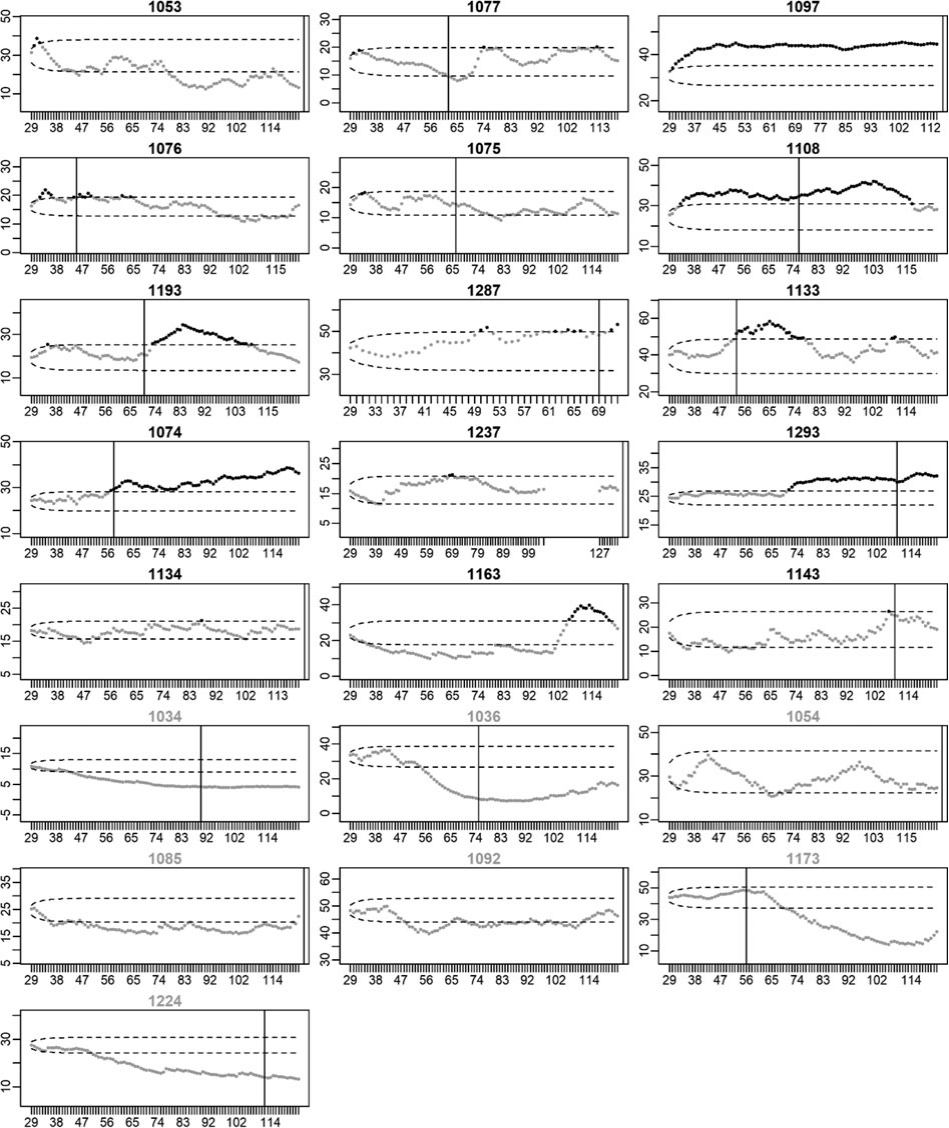
EWMA of restlessness for participants with a recurrence of depression. *Note:* The *x*-axis denotes the time in days. The *y*-axis denotes the EWMA of restlessness. Dots represent the EWMA at the day indicated on the *x*-axis. Dots are colored black when the EWMA exceeded the UCL indicated by the upper dashed line; dots are gray otherwise. Black titles indicate participants for whom the EWMA exceeded the UCL (true positives), titles are gray otherwise (false negatives). The vertical black lines indicate the start of the increase in depressive symptoms. When recurrence of depressive symptoms occurred after the final EMA observation, the black line is printed at the end of the EMA period.

**Fig. 4.**
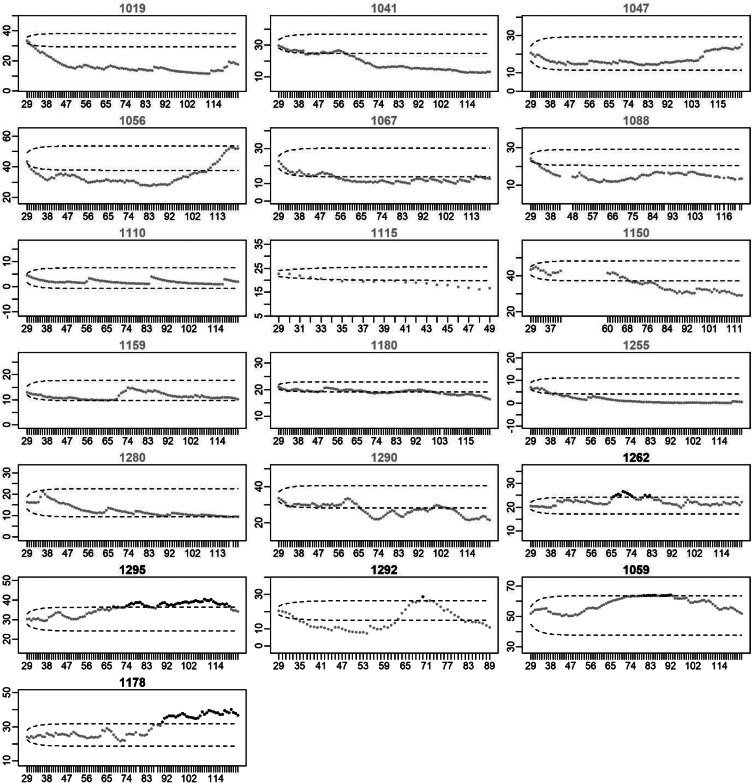
EWMA of restlessness for participants who stayed in remission. *Note:* The *x*-axis denotes the time in days. The *y*-axis denotes the EWMA of restlessness. Dots represent the EWMA at the day indicated on the *x*-axis. Dots are colored black when the EWMA exceeded the UCL indicated by the upper dashed line; dots are gray otherwise. Gray titles indicate participants for whom the EWMA did not exceed the UCL (true negatives), titles are black otherwise (false positives).

Figures 3 and 4 present the EWMA control charts of restlessness for each participant individually. When inspecting these graphs visually, it can be seen that each participant has their own personalized control limits, which were exceeded for a longer period of time, briefly, or not at all. The figures show that the size and duration of significant increases in the EWMA of restlessness varied between individuals.

### Increases in restlessness as an early sign of recurrence of depression

In 15 of the 22 participants with a recurrence, an increase in restlessness was found (i.e. sensitivity = 68.2%). This was far more than the five false positives that were found in the 19 participants that stayed in remission (i.e. specificity = 73.7%). A post-hoc Fisher's exact test showed that the percentage of true positives was significantly higher than the percentage of false positives (*p* = 0.01). Of the 20 participants in which an increase in restlessness was detected, 15 also experienced an increase in depressive symptoms, yielding a PPV of 75.0%. Of the 21 participants without an increase in restlessness, 14 did not experience an increase in depressive symptoms during the study period, yielding a NPV of 66.7%.

In 14 of the 15 cases (93.3%), the increase in restlessness could be found before the first possible day the increase in depressive symptoms could have happened. For one of the 15 participants, the EWMA exceeded the UCL on the first day of the week in which the increase in depressive symptoms occurred. The increase in restlessness was detected at least 1 week before recurrence in 12 of the 15 cases (80.0%) and at least 1 month before the first day recurrence was noticed in 10 of the 15 cases (66.7%).

In a subset of the participants who did not show an increase in restlessness, a decrease in restlessness was found. A decrease in restlessness was detected in seven of the 22 participants with a recurrence (31.8%) and in 11 of the 19 participants who stayed in remission (57.9%).

### Sensitivity analyses

Sensitivity analyses yielded highly similar results. Re-running the analyses without Winsorizing the data did not change the results (sensitivity = 68.2%, specificity = 73.7%). Re-running the analyses using only the 14 participants for whom the rise in depressive symptoms occurred during the EMA period yielded a sensitivity of 71.4% and a specificity of 73.7%. Re-running the analyses using the raw data instead of day averages of restlessness yielded a sensitivity of 72.7% and a specificity of 73.7%.

## Discussion

This study showed that increases in mean levels of restlessness could be detected in real-time using EWMA control charts in the majority of the participants with a recurrence of depressive symptoms. Rises in restlessness never occurred after recurrence of depressive symptoms, indicating that restlessness is an early sign of depressive symptom recurrence. In about two-thirds of the participants who experienced an increase in restlessness before the recurrence of depression, this increase could be detected more than a month before the onset of the core symptoms of depression.

The findings suggest that restlessness is a common prodromal symptom of depression. This is in line with previous studies showing that feelings of anxiety, including symptoms related to restlessness, such as irritability and tension, are often reported in the prodromal phase of depression (Benasi et al., 2021). Restlessness may be an indicator of hyperarousal or disturbed activity of the sympathetic and parasympathetic nervous system. Changed autonomic nervous activity, for example, in the form of blunted heart rate reactivity in response to stress and increased muscle sympathetic nervous activity, has been shown to be associated with depression (Arias et al., 2020; Scalco et al., 2009; Schiweck et al., 2019). A restless state that is characterized by disturbed autonomic nervous activity and hyperarousal may come along with symptoms such as sleep problems, rumination, and reduced attentional control, which all have been shown to predict depression (Blake et al., 2018). In line with this, symptoms of hyperarousal, such as irritability, sleep problems, tension, and somatic complaints, as well as symptoms of exhaustion (fatigue and reduced energy) were found to be among the most commonly reported prodromal symptoms of depression (Benasi et al., 2021). The current study adds to this by demonstrating that within-person increases in restlessness often precede the recurrence of depression.

However, we can only speculate about the mechanisms explaining the current findings as this study did not provide insight in mechanisms nor the causes for the rises in restlessness. It could, for example, be that antidepressant discontinuation led to increased restlessness in some participants, which in turn may have increased the probability of recurrence of depression. Even if this is the case, all participants in the sample discontinued their antidepressant medication while increases in restlessness were mostly found in the group with a recurrence of depression, supporting the conclusion that increases in restlessness signal recurrence of depression. Regardless of the causes of rises in restlessness, being able to detect an upcoming increase in depressive symptoms using restlessness scores is relevant in and of itself, even if the precise mechanism is not yet fully understood.

The current study shows that EWMA control charts are relatively accurate in foreseeing recurrence of depression by detecting within-person increases in mean levels of restlessness. The sensitivity (68.2%) and specificity (73.7%) of the current idiographic risk assessment based on monitoring of restlessness using the EWMA procedure was comparable to, or slightly higher than, state-of-the-art models aiming to predict recurrence of depressive symptoms based on cross-sectional patient characteristics (Judd, Schettler, & Rush, 2016; Klein et al., 2018; van Loo, Bigdeli, Milaneschi, Aggen, & Kendler, 2020). As cross-sectional data have been collected and analyzed for decades, whilst EMA time-series have only been collected on a larger scale since the introduction of the smartphone, we consider achieving similar results in this early stage promising. Moreover, the results show that two commonly used cross-sectional predictors of recurrence (i.e. residual symptoms and number of previous discontinuation attempts), as well as absolute levels of restlessness were not significantly related to recurrence of depression in the current sample. Therefore, we can conclude that within-person increases in restlessness were a better predictor of recurrence than any one of these baseline characteristics in the current sample. The effectiveness of combining the EWMA control chart with existing cross-sectional prognostic tools could be investigated in future research.

An advantage of the EWMA method is that exclusively within-person change over time was used to foresee the recurrence of depressive symptoms. This implies that the EWMA procedure can be applied to EMA data of a single individual to assess an increase in the risk of recurrence without the need for comparing these EMA data to the data of anyone else. Another advantage of the current idiographic risk assessment over these cross-sectional methods is that the EWMA control chart provides information on when someone's risk of recurrence starts to increase compared to themselves (i.e. a within-person increase in risk). At one moment in time, no increase in restlessness may be detected while a couple of weeks later, restlessness may have increased, indicating a within-person increase in the risk of recurrence. Although this elevated risk cannot be used to predict when depressive symptoms will recur specifically, it may help patients and clinicians to decide when action should be taken. That is, interventions aimed at prevention of recurrence of depression may only be needed when a within-person increase in restlessness is detected and make little sense when this is not the case as there is no indication this individual's risk is elevated. Patients could be alerted of elevated risk between 1 week and 5 months before recurrence of depression based on the EWMA control chart of restlessness in the current sample. For two-thirds of the patients the alert came more than a month in advance, providing patients and clinicians sufficient time to intervene and potentially prevent recurrence of depression.

It should be noted that instead of an increase in restlessness, a decrease in restlessness was detected in the participants with a recurrence of depressive symptoms who did not have an increase in restlessness and in more than half of the participants who stayed in remission. The downward trend may have been caused by the phenomenon of an ‘initial elevation bias’, which has been shown to occur in studies using repeated measurements of negative mental states over time (Shrout et al., 2018). This, however, did not affect the finding that the majority of the participants with a recurrence showed an increase in restlessness while such an increase was found in only a minority of the participants who stayed in remission.

Furthermore, the data of the current study were not gathered with the aim of investigating the EWMA procedure, and participants were already tapering antidepressant medication during phase I of the study. As a result, we cannot assume that the process was in control (i.e. no change in restlessness) during each participant's phase I period (baseline). In cases where restlessness was already increasing during phase I, the EWMA procedure would have been less sensitive for detecting similar changes during phase II, which may have led to an increase in the number of false negatives. Future studies aiming to use the EWMA procedure should consider planning phase I data collection ahead, as this could increase the sensitivity and accuracy of the procedure beyond the results obtained in the current study.

A strength of the current study was that the out-of-sample performance of the method used in Smit et al. (2019) was tested confirmatively. Therefore, it is more likely that the results will also generalize to new samples of patients discontinuing their antidepressant medication. Another strength of the study was the median of 540 observations for each individual patient in the study, yielding a total of 21 180 separate observations. A limitation was the small amount of between-person data (*N* = 41), which limit generalizability, although it should be noted that the sample size is fairly large for a replicated single-subject time-series study. Another limitation of the study was that restlessness was measured with one item and we could therefore not assess the psychometric properties of our measurement of restlessness. Furthermore, because of the adopted study design, the results may only generalize to patients who discontinue their antidepressant medication, who are highly educated and motivated to engage in ambulatory assessments for a prolonged period of time, and who experience recurrence of depressive symptoms between 2 weeks and 5 months after discontinuation of antidepressant medication. Finally, future research may provide insight in the effect of the width of the control limits, the tuning parameter *λ*, and length of the observation period on the percentage of true positives and false positives.

Although the potential of self-monitoring to detect early signs of recurrence ahead of time has been recognized (Bos et al., 2019; Busk et al., 2020), it had so far not been realized. We demonstrate that by applying the EWMA control chart to self-monitoring data of restlessness, the rise of a prodrome can be detected in real-time, and an assessment of an individual's current risk of depressive symptoms recurrence can be made. Future research may examine whether the predictive value of the EWMA chart could be improved by monitoring multiple prodromal symptoms simultaneously. As only a few studies investigated within-person change over time prior to the recurrence of depression, it is likely there is substantial room for improvement in this field of research.

## Footnotes

† The notes appear after the main text.

## Notes

1 This is the same number of days that is obtained when using *λ*=0.05 and *L*=3 in the raw data, which are the settings used in Smit et al. (2019). Correcting the *L* parameter when using day averages is needed to achieve a sensitivity and specificity that accurately replicates Smit et al. (2019), as without this correction the sensitivity would be underestimated, and the specificity would be overestimated. Due to a statistical error, the preregistration of this study did not implement this correction. We corrected this statistical error in the current manuscript.
